# Digital support and artificial intelligence in cancer patients undergoing radiation therapy: patient utilization, acceptance and attitudes

**DOI:** 10.3389/fonc.2025.1546221

**Published:** 2025-09-26

**Authors:** Franziska Springer, Peter Kurt Hambsch, Anja Mehnert-Theuerkauf, Nils Henrik Nicolay

**Affiliations:** ^1^ Department of Medical Psychology and Medical Sociology, Comprehensive Cancer Center Central Germany (CCCG), University Medical Center, Leipzig, Germany; ^2^ Department of Radiation Oncology, Comprehensive Cancer Center Central Germany (CCCG), University Medical Center, Leipzig, Germany

**Keywords:** cancer, digital support tools, artificial intelligence, cancer care, radiation therapy

## Abstract

**Background:**

New technologies, such as digital support tools or artificial intelligence (AI) in cancer diagnostics and treatment, offer new possibilities for cancer care. Evidence on patients’ attitudes towards new technologies within the context of cancer care is however very limited to date. We aimed to investigate utilization, acceptance and attitudes towards digital support tools and AI within the context of cancer treatment and to identify associated patient-related factors.

**Methods:**

This exploratory observational cross-sectional study assessed adult cancer patients undergoing radiation therapy with a set of validated questionnaires in addition to newly developed items for this study on acceptance and attitudes towards new technologies within cancer care. Utilization, acceptance and attitudes towards new technologies were assessed descriptively and the impact of associated factors was analyzed using logistic regression models and Analysis of Variance (ANOVA).

**Results:**

In total, 154 cancer patients were included in our study with a mean age of 63.7 years, 51% were male. In general, patients felt inadequately informed about new technologies, with only 12% feeling informed about digital support tools and 16% feeling informed about AI within their cancer care. One in two patients had used digital support (e.g. websites, apps, wearables). The majority perceived digital support tools as beneficial for all ages (65%) and were open towards the use of AI within their healthcare (79%). Nevertheless, patients reported a strong preference for in-person care, and some patients indicated concerns about possible mistakes by AI (27%). Trust in new technologies revealed a mixed pattern, whereby older patients, those with lower socioeconomic resources, and limited digital health literacy (DHL), were less likely to use digital support (age: *p* = 0.001, socioeconomic: *p* = 0.002, DHL: *p* < 0.001) and reported lower trust in new technologies (age: *p* = 0.01, socioeconomic: *p* < 0.001, DHL: *p* < 0.001) for their cancer care.

**Conclusion:**

While patients are generally open to the use of AI in healthcare, their concerns underscore the need for future research into the physician’s role in ensuring its responsible, safe, and patient-centered utilization. Strengthening DHL, improving information provision, and reducing access barriers for vulnerable groups may enable a more effective integration of new technologies into routine cancer care.

## Introduction

Digital advancements and new technologies have significantly impacted cancer care, encompassing tools for the collection and utilization of health data in prevention, diagnosis, treatment, and follow-up care. Digital support tools, such as apps, websites, or wearables aim to support patients in managing the disease and its potential side effects, and provide flexible, individualized support regardless of time and location. Such tools are already widely used to improve patient-centered clinical cancer care (1–5). However, the rapid expansion of these digital tools presents challenges for many patients and healthcare providers (6–9). These challenges include a lack of knowledge about the functionality and benefits of new technologies, concerns about data privacy and security, an overwhelming amount of information that can lead to confusion, and difficulties in effectively using the data for medical treatment. Moreover, the widespread use of digital support tools may exacerbate social and health inequalities, as not all patients are able to access these tools (10), or have sufficient digital health literacy (DHL) to use them effectively (11). But also advancements in artificial intelligence (AI) are increasingly used in oncological care for diagnostic procedures as well as treatment planning, for example analyzing laboratory results, interpreting medical images, or developing personalized therapeutic plans (12–14).

To date, there has been limited research on the utilization and acceptance of digital support tools for cancer patients (15–21). In addition, patients’ perceptions and trust regarding the use of AI in oncological treatment is largely unknown despite the availability of applications for AI in cancer care, such as in the fields of radiology, dermatology or radiation oncology. Recent studies generally indicate a high level of patient acceptance regarding digital support, such as video consultations, digital therapeutics, and digital symptom monitoring, along with a willingness to continue using these tools in the future (15–21). However, a high variability remains among existing studies, as noted in a systematic review on digital symptom monitoring in cancer patients (22). It is important to note that the level of acceptance of digital tools may vary considerably across different patient populations, depending on factors such as age, digital literacy, access or prior exposure to such technologies. In addition, while the majority of patients are open to the use of AI in cancer treatment, particularly when combined with an evaluation of the treating oncologist, many still express concerns about potential errors made by AI and the possible replacement of in-person care (19, 23–27).

In summary, the current data on the utilization, acceptance and attitudes towards digital support tools and AI among oncological patients is limited, and some studies in this rapidly evolving field are already outdated. It remains unclear to what extent patients trust new technologies within the context of cancer diagnosis and care, how frequently digital support tools are used, what reasons exist for their use or non-use, and how well patients are informed about AI in cancer treatment and to what extent they trust its use in cancer treatment. For an effective integration of new technologies into cancer care, it is essential to identify patient attitudes and potential barriers. Therefore, the aim of this exploratory study was to assess the utilization, acceptance and attitudes toward digital support tools and AI in the context of cancer and its treatment, as well as to identify influencing factors, such as age or DHL. This may help inform future implementation strategies for digital technologies and guide policy decisions regarding the routine use of digital support tools and AI in cancer care.

## Materials and methods

### Study design and sample

We conducted an observational cross-sectional study in the Department of Radiation Oncology in collaboration with the Department of Medical Psychology and Medical Sociology, Comprehensive Cancer Center Central Germany, University Medical Center Leipzig. The target population was subject to the following inclusion criteria: (1) a cancer diagnosis according to ICD-10 (C00-C96), (2) age ≥ 18 years, (3) sufficient German language skills to answer the questionnaire, and (4) currently undergoing radiotherapy at the Cancer Center. Patients who were physically or mentally unable to participate were excluded from the study. The present study aims to investigate a patient population that has been exposed to the use of AI in cancer treatment, that is patients undergoing radiotherapy at the study center where AI is routinely integrated into radiotherapy treatment. AI is employed to support specialists in radiotherapy planning (contouring of radiation volumes) and, for some patients, in adaptive radiotherapy. All AI-assisted steps are reviewed and, if necessary, manually corrected by specialists. When AI is used in the actual delivery of radiotherapy, patients are required to provide written informed consent. Beyond this primary rationale for the selection of our study population, these patients also represent a relevant target group for digital support tools, such as those designed to monitor physical and psychological side effects or to promote self-management. The study was approved by the local ethics committee of the Medical Faculty, University of Leipzig (418/23-ek).

### Data collection

Patients in the outpatient unit of the Department of Radiation Oncology at the University Medical Center Leipzig were routinely informed about the study between June and October 2024, resulting in the accessible population of 646 patients. Patients received written information about the study by trained nurses during their routine hospital admissions. After providing written informed consent, patients were asked to complete a paper-pencil questionnaire while they were waiting. Due to the clinical routine and individualized procedures at the hospital, it could not be ensured that all patients treated during the recruitment period received study information. As a result it was not possible to calculate a valid response-rate.

### Measurement

Sociodemographic information was collected through self-reporting in the questionnaire, i.e. age, gender, income, employment, partnership status. Medical data were obtained from the hospital information system, i.e. diagnosis, date of diagnosis, state of recurrence.

Several validated instruments were used. Psychological distress was assessed with the one-item Distress Thermometer (DT) on a scale from 0 (no distress) to 10 (extreme distress), with a validated cut-off ≥ 5 indicating clinically relevant distress (28), as well as the four-item PHQ-4 that captures symptoms of anxiety and depression and has been validated in this clinical setting (cut-off ≥ 6) (29). The use of additional psychosocial support was assessed with the SozU (30). DHL was measured with the validated eHealth Literacy Scale (eHEALS) (31), an eight-item instrument, rated on a scale from 1 (strongly disagree) to 5 (strongly agree). Scores from 8–20 indicate low, 21–30 medium, and 31–40 high DHL.

Due to the lack of validated instruments, the utilization, acceptance and attitudes towards digital support tools and AI were measured using items developed specifically for this study. All items (German original and English translation) are presented in the **Appendix**. Multiple-choice answers, including open-ended options, were used to assess digital support tool utilization, the reasons for their use or non-use, and preferences for digital support or in-person care. The patients’ trust in new technologies was assessed on a scale from 0 (not at all) to 10 (very much). Acceptance and attitudes towards AI and digital support tools were assessed through various statements on a 5-point Likert scale (1=strongly disagree, 2=disagree, 3=neutral, 4=agree, 5=strongly agree). The item development was guided by discussions within a multi-professional research team with expertise in digital support tools and AI in healthcare, ensuring content validity. To refine item clarity and comprehension, the items were pretested in a small pilot study with a sample from the target population. The aim of this study was not to develop a validated questionnaire on attitudes towards AI and digital support, but rather to present initial exploratory findings on this topic. As the analysis will be conducted on a single-item basis, psychometric assessments are not applicable. Consequently, the evaluation of reliability is limited.

### Patient involvement

The newly designed items for the study were tested for their comprehensibility and usefulness in terms of early patient participation in a preliminary piloting phase. This involved testing the preliminary questionnaire with five patients from the Department of Radiation Oncology and subsequently conducting qualitative interviews with these patients. Based on the findings of this phase, the items were modified as necessary. The responses of the pilot patients were not included in the final analysis.

### Statistical analysis

Sociodemographic and medical characteristics of the sample were presented using descriptive statistics. The utilization and acceptance of digital support and AI in the context of cancer were summarized descriptively through mean values, frequencies and 95% confidence intervals (CI). Deviations from the full sample size represent the number of missing values. Acceptance and attitudes towards AI and digital support were reported as the frequency distribution of responses for each statement.

In order to test our hypotheses that sociodemographic and medical factors (age, gender, education, partner, distress, DHL, time since diagnosis, relapse and additional psychological support) significantly predict the utilization of digital support tools and the level of trust in new technologies, we applied logistic regression models, t-tests and ANOVAs. The analyses regarding the newly develop items on acceptance and attitudes towards digital support tools and AI were exploratory. Data analysis was conducted using R, version 4.3.1 (32). Level of significance was set at α=5%.

## Results

We enrolled 154 patients in our study, half of them were female (49%) with a mean age of 63.7 years (**Table 1**). All patients were currently undergoing radiation therapy. The most common diagnoses were breast, prostate, colorectal and lung cancer. More than half of the participants were retired (61%) and lived in a relationship (73%). Overall, 53% of the participants reported psychological distress above the clinical cut-off on the DT (≥ 5), and 55% on the PHQ-4 (≥ 6). DHL was low, medium, and high in 14%, 55%, and 31% of the participants, respectively.

**Table 1:** Sample characteristics (n=154).

Variable	N	(%)
Age, mean (SD), range	63.7 (12.1), 30-85	
Gender		
Female	75	(49)
Male	79	(51)
Family situation		
Single	22	(15)
Married	102	(69)
Divorced	12	(8)
Widowed	12	(8)
Partner	98	(73)
Education		
Secondary School	82	(56)
High School	29	(20)
University	36	(24)
Monthly income		
≤ 2000€	86	(63)
> 2000€	50	(37)
Employment		
Currently working	46	(31)
Unemployed	4	(3)
Retired (old-age, disability)	90	(61)
Other	8	(5)
Diagnosis		
Breast	54	(35)
Prostate	34	(22)
Colorectal	13	(8)
Lung	8	(5)
Gynecological	8	(5)
Other	37	(24)
Months since diagnosis, Median (SD)	27 (26.1), 0-151	
Relapse	25	(16)
Utilization of psychological support	24	(21)

Percentages are based on valid responses, and the difference in n to the total sample therefore corresponds to missing values.

Most patients reported owning a mobile device (95% [CI: 92-98%], n=146), with 46% [CI: 38-54%] (n=67) of them using it at least partially for managing their health. So far, 12% [CI: 7-17%] (n=18) had used telehealth services, but 71% [CI: 64-78%] (n=103) indicated they would be open to digitally share their medical data with healthcare professionals. Patients’ trust in new technologies (telemedicine, AI, etc.) for their own healthcare revealed a mixed pattern on a scale from 0 (not at all) to 10 (very much), with a mean value of 5.2 (SD = 3.2, CI: 4.7-5.7). Notably, 22% [CI: 15-29%] (n=31) reported values ≤2, indicating low trust in new technologies.

### Digital support tools within the context of a cancer disease

Half of the participants had used digital support tools in the past (51% [CI: 43-59%], n=79), but only 12% [CI: 6-18%] (n=15) felt adequately informed about available digital support tools for cancer patients. The most common reasons for using digital support tools were to obtain information about the cancer diagnosis and its treatment, to receive support with managing physical symptoms, and to get lifestyle recommendations. The primary reason for patients not using digital support tools was a preference for in-person care. Participants indicated a strong preference for in-person support as opposed to digital support when dealing with physical (69% [CI: 61-77%], n=97) and psychological symptoms (70% [CI: 62-78%], n=91).

**Figure 1:**
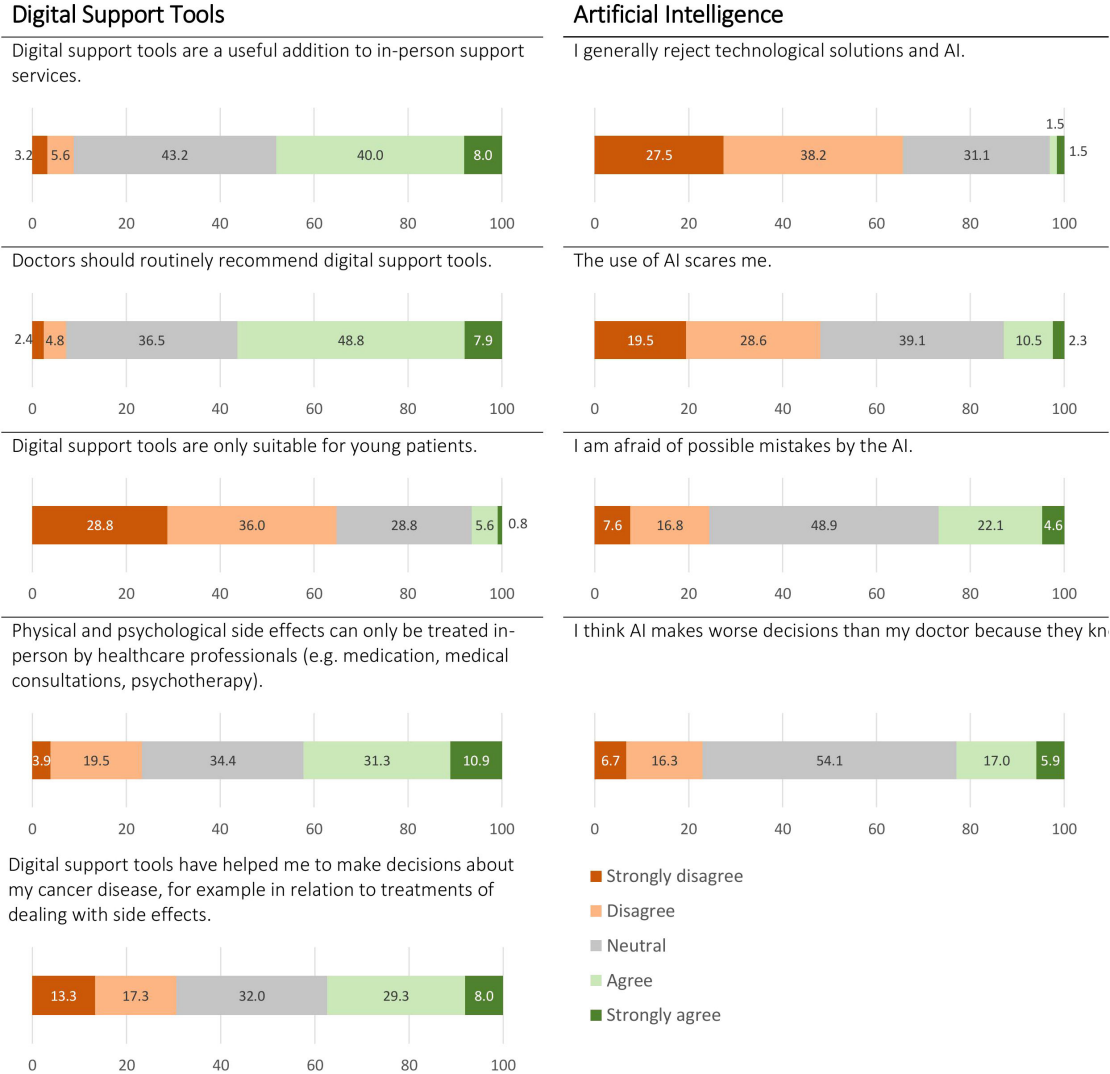
Attitudes towards digital support tools and artificial intelligence (AI) in cancer patients undergoing radiation therapy.

Attitudes toward digital support tools are displayed in **Figure 1**. The majority of patients indicated that digital support tools could be a valuable addition to in-person care (48% [CI:39-57%], n=60), that doctors should routinely recommend them (56% [CI: 47-65%], n=71), and that they could be beneficial for patients of all ages (65% [CI: 57-73%], n=81). However, 42% [CI: 33-51%] (n=54) felt that physical and psychological late effects could only be treated effectively in-person by healthcare professionals. Of the patients who had used digital support tools, 37% [CI: 26-48%] (n=28) reported that it had helped them in decision-making related to their treatment and managing side effects.

### Artificial intelligence during radiation therapy

Overall, only 16% [CI: 10-22%] (n=21) of patients felt adequately informed about the use of AI in their radiation therapy, while 79% [CI: 72-86%] (n=104) would agree to the use of AI in their treatment. Openness towards AI was high with 65% [CI: 57-73%] (n=92) indicating they have most trust in a combination of a doctor and AI for their treatment and the interpretation of lab results. Notably, no patient reported trusting AI alone.

**Figure 1** displays patients’ attitudes towards AI. A mere 3% [CI: 0-6%] of patients (n=4) indicated that they generally reject technological solutions and AI. However, 13% [CI: 7-19%] (n=17) maintained that the use of AI scares them, 27% [CI: 19-35%] (n=35) stated that they were afraid of possible mistakes by AI, and 23% [CI: 16-30%] (n=31) believed that AI would make worse decisions than their treating doctors, as the latter know better what is right for their patients.

### Associated factors with utilization and trust in new technologies

Older patients (≥65 years), patients with lower education (secondary school), with low or medium DHL, and those without additional psychological support were less likely to use digital support tools (**Table 2**). In addition, lower trust in new technologies (telemedicine, AI, etc.) for their healthcare was observed in older patients (≥65 years), patients with lower education (secondary school), patients with low or medium DHL, and those without additional psychological support. No further sociodemographic and medical factors were associated with utilization and trust in new technologies (all *P*>.05).

**Table 2:** Sociodemographic and medical factor associated with utilization and trust in new technologies.

Variable	Utilization of digital support tools		Trust in new technologies	
	β (SE)	*P*	t/F (df)	*P*
Age (<65 *vs*. ≥65)	1.08 (0.34)	**0.001**	2.71 (142)	**0.01**
Gender (men *vs*. women)	0.47 (0.32)	0.15	-1.10 (139)	0.27
Education			10.68 (2)	**<0.001**
Secondary *vs*. University	-1.32 (0.43)	**0.002**		**<0.001**
High school *vs*. University	0.14 (0.55)	0.79		0.07
Partner	0.36 (0.39)	0.36	-0.54 (58)	0.59
Psychological distress (DT)	0.02 (0.07)	0.79	0.67 (132)	0.50
Digital health literacy (eHEALS)			9.22 (2)	**<0.001**
Low *vs*. high	-2.67 (0.69)	**<0.001**		**<0.001**
Medium *vs*. high	-1.28 (0.46)	**0.01**		**0.047**
Utilization of additional psychological support	1.50 (0.59)	**0.01**	-2.2 (39)	**0.03**
Time since diagnosis (months)	0.00 (0.01)	0.90	0.87 (142)	0.39
Relapse	0.83 (0.46)	0.07	-0.26 (34)	0.80

Distress Thermometer (DT), eHealth Literacy Scale (eHEALS); significant values marked in bold.

## Discussion

This explorative observational study shows that the majority of participating cancer patients – particularly younger patients, those with higher educational levels and with greater DHL – are open to new technologies in cancer care, including digital support and AI. However, only a small proportion felt adequately informed about the potential use of AI, available digital tools and their application in cancer care. One in two participating patients had previously used digital support tools such as websites, apps or wearables in managing their cancer disease, and four out of five patients would agree to the use of AI for their treatment. Nonetheless, a subgroup of participating patients remains wary, with low trust in new technologies and concerns about possible mistakes associated with AI.

Especially in technological sub-specialties of modern oncology such as radiology and radiation oncology, AI-based diagnostic or treatment support tools are available and used for routine care. In the context of radiotherapy, AI-aided treatment planning and real-time treatment adaptation (“plan of the day”) have been established and demonstrated benefits for patients in early clinical trials (33–35).

Our findings on the high level of patient acceptance of digital support align with the few existing studies on attitudes toward digital support tools, such as video consultations, digital therapeutics, or digital symptom monitoring (15–21). These tools are perceived as beneficial across age groups, indicating their broad applicability. However, they are currently used predominantly by younger patients. With the rapid increase in available digital support tools (36, 37), older patients and those with limited DHL may face challenges in finding appropriate and effective tools for their individual needs and using them effectively for supportive care (38, 39). This is supported by a scoping review that identifies key facilitators for telemedicine use, such as convenience of use, perceived availability, physicians’ recommendations, and patients’ technical knowledge (18).

Although only a small proportion of participating patients felt adequately informed about the potential use of AI, four out of five patients expressed willingness to agree to its use in their cancer treatment, a number consistent with previous studies (19, 23–27). Currently, many healthcare professionals lack the training to understand or explain the applicability of AI algorithms in cancer care and may struggle with understanding these algorithms themselves. Despite the limited information provided to patients about AI, its broad acceptance may be influenced by growing media coverage. Nonetheless, some participating patients remain cautious, citing concerns such as fear of AI or potential errors, which underline their strong preference for in-person care and human monitoring of AI applications (40). This skepticism is reflected in the few existing studies (25–27), underscoring the need for enhanced public education on AI along with continued human involvement and monitoring in its application. In-person care will likely remain central and cannot be replaced by technical solutions alone.

Our findings reveal digital health disparities in the use and trust of new technologies, with disadvantaged groups comprising older patients, those with lower educational levels, and patients with limited DHL. This underscores the existence of a digital divide – the gap between individuals who have access to and benefit from technological solutions and those who do not (38, 41). Socioeconomic disadvantages and limited DHL have previously been associated with poorer physical and mental health outcomes in cancer care (10, 11, 42). Therefore, it is essential for healthcare professionals to consider these factors when providing information about digital support, tailoring it to meet the needs of vulnerable subgroups.

Across multiple items on acceptance and attitudes towards new technologies, patients frequently selected the response option “neutral”, reflecting ambivalence or uncertainty. This may either be explained by central tendency bias, or by limited exposure to or understanding of these technologies, and a need for more information. Such patterns highlight the importance of patient education and communication when implementing digital innovations in cancer care.

### Clinical implications

The findings of this study reveal a substantial gap in patient knowledge about digital support tools and AI, emphasizing the urgent need for enhanced informational resources and the better integration of these topics into clinical communication. While certain patient groups may already benefit from using digital support tools, finding them accessible and helpful, access remains challenging for others – particularly older adults, those with limited digital literacy, and individuals with lower educational backgrounds. To address and reduce digital health disparities, it is essential to adapt and tailor digital support tools to meet the needs of underserved populations. Additionally, increasing patient knowledge and awareness about existing applications and the role of AI in cancer diagnostics and treatment may foster greater acceptance and potentially improve treatment responses (43) and adherence (44). Finally, healthcare providers should address patients’ concerns about AI openly, as such discussions can help mitigate unrealistic fears and encourage a more positive perception of these technologies in clinical settings.

### Strengths and limitations

This study is among the first investigations on acceptance and attitudes towards digital support for cancer patients and the use of AI during cancer treatment. It constitutes the first assessment of patient feelings towards AI in the context of radiotherapy where AI is currently broadly available for target volume delineation and contouring of organs-at-risk as well as real-time online adaptation of radiation treatment plans. We used validated clinical information alongside established tools to assess DHL and psychological distress. However, several limitations warrant consideration. The questionnaire used in this study was exploratory in nature and not designed for formal psychometric validation. As such, items are often close-ended, and some items may be subject to unclear or ambiguous wording. These limitations may have influenced how participants interpreted and answered certain items and should be taken into account when interpreting the findings. The inclusion of self-developed items, however, was necessary due to the innovative nature of the research, as there is currently no validated assessment tool for the utilization and acceptance of digital support and AI. The need for such a tool is evident to enhance the comparability of results in future studies. Additionally, the small sample size, monocentric nature of the study, and brief data collection period may introduce bias and limit the generalizability of our findings. We were unable to conduct non-responder analyses and calculate a response-rate, which may have implications for the comprehensiveness of our findings. However, the sociodemographic and medical characteristics of our sample, such as age, gender and tumor types, are largely consistent with data from the German cancer registry (45), and thus indicate a broadly representative sample. Lastly, our findings pertain the context of radiation oncology, and their generalizability to other fields of oncology remains to be demonstrated. Future research should explore diverse populations to broaden the applicability of the results.

## Conclusion

Cancer patients are generally open to digital support and the use of AI as part of their cancer treatment, but available digital tools are mainly younger, educated and digitally literate patients who make use of such tools. There is generally a strong preference for in-person care and human monitoring of AI applications, thus representing an irreplaceable part of comprehensive cancer care. Our results further underscores the need to clearly define physicians’ responsibilities when AI is routinely used in healthcare, including their critical role in communicating its use, benefits, and limitations to patients. To ensure effective integration of new technologies into routine cancer care, information provision needs to be improved and barriers to access for vulnerable groups need to be reduced.

## Data Availability

The raw data supporting the conclusions of this article will be made available by the authors, without undue reservation.

## References

[1] Janssen A Brunner M Keep M Hines M Nagarajan S Kielly-Carroll C. Interdisciplinary eHealth practice in cancer care: A review of the literature. IJERPH. (2017) 14:1289. doi: 10.3390/ijerph14111289, PMID: 29068377 PMC5707928

[2] Penedo FJ Oswald LB Kronenfeld JP Garcia SF Cella D Yanez B. The increasing value of eHealth in the delivery of patient-centred cancer care. Lancet Oncol. (2020) 21:e240–51. doi: 10.1016/S1470-2045(20)30021-8, PMID: 32359500 PMC7643123

[3] Gussoni G Ravot E ZecChina M Recchia G Santoro E Ascione R. Digital therapeutics in Oncology: findings, barriers and prospects. A narrative review. Ann Res Oncol. (2022) 02:55. doi: 10.48286/aro.2022.39

[4] Springer F Maier A Friedrich M Raue JS Finke G Lordick F. Digital therapeutic (Mika) targeting distress in patients with cancer: results from a nationwide waitlist randomized controlled trial. J Med Internet Res. (2024) 26:e51949. doi: 10.2196/51949, PMID: 38663007 PMC11082740

[5] Springer F Mehnert-Theuerkauf A. Content features and its implementation in novel app-based psycho-oncological interventions for cancer survivors: a narrative review. Curr Opin Oncol. (2022) 34:313–9. doi: 10.1097/CCO.0000000000000836, PMID: 35837701

[6] Sim I. Mobile devices and health. New Engl J Med. (2019) 381:956–68. doi: 10.1056/NEJMra1806949, PMID: 31483966

[7] Ellis LA Meulenbroeks I Churruca K Pomare C Hatem S Harrison R. The application of e-mental health in response to COVID-19: scoping review and bibliometric analysis. JMIR Ment Health. (2021) 8:e32948. doi: 10.2196/32948, PMID: 34666306 PMC8651237

[8] El Shafie RA Weber D Bougatf N Sprave T Oetzel D Huber PE. Supportive care in radiotherapy based on a mobile app: prospective multicenter survey. JMIR Mhealth Uhealth. (2018) 6:e10916. doi: 10.2196/10916, PMID: 30166275 PMC6137282

[9] Janssen S El Shafie RA Ruder AM Buergy D Scafa D Giordano FA. Mobile applications in radiation oncology-current choices and future potentials. Strahlenther Onkol. (2023) 199:337–49. doi: 10.1007/s00066-023-02048-y, PMID: 36810957 PMC9943039

[10] Fareed N Swoboda CM Jonnalagadda P Huerta TR. Persistent digital divide in health-related internet use among cancer survivors: findings from the Health Information National Trends Survey, 2003–2018. J Cancer Surviv. (2021) 15:87–98. doi: 10.1007/s11764-020-00913-8, PMID: 32671557 PMC7360998

[11] Kemp E Trigg J Beatty L Christensen C Dhillon HM Maeder A. Health literacy, digital health literacy and the implementation of digital health technologies in cancer care: the need for a strategic approach. Health Prom J Aust. (2021) 32:104–14. doi: 10.1002/hpja.387, PMID: 32681656

[12] Bhinder B Gilvary C Madhukar NS Elemento O. Artificial intelligence in cancer research and precision medicine. Cancer Discov. (2021) 11:900–15. doi: 10.1158/2159-8290.CD-21-0090, PMID: 33811123 PMC8034385

[13] Huang S Yang J Fong S Zhao Q. Artificial intelligence in cancer diagnosis and prognosis: Opportunities and challenges. Cancer Lett. (2020) 471:61–71. doi: 10.1016/j.canlet.2019.12.007, PMID: 31830558

[14] Bi WL Hosny A Schabath MB Giger ML Birkbak NJ Mehrtash A. Artificial intelligence in cancer imaging: Clinical challenges and applications. CA A Cancer J Clin. (2019) 69:127–57. doi: 10.3322/caac.21552, PMID: 30720861 PMC6403009

[15] Kwon M Jung Y-C Lee D Ahn J. Mental health problems during COVID-19 and attitudes toward digital therapeutics. Psychiatry Investig. (2023) 20:52–61. doi: 10.30773/pi.2022.0150, PMID: 36721886 PMC9890043

[16] Nurtsch A Teufel M Jahre LM Esber A Rausch R Tewes M. Drivers and barriers of patients’ acceptance of video consultation in cancer care. DIGITAL Health. (2024) 10:20552076231222108. doi: 10.1177/20552076231222108, PMID: 38188860 PMC10768612

[17] Vogel MME Eitz KA Combs SE. Web-based patient self-reported outcome after radiotherapy in adolescents and young adults with cancer: survey on acceptance of digital tools. JMIR Mhealth Uhealth. (2021) 9:e19727. doi: 10.2196/19727, PMID: 33427669 PMC7834941

[18] Pang N-Q Lau J Fong S-Y Wong CY-H Tan K-K. Telemedicine acceptance among older adult patients with cancer: scoping review. J Med Internet Res. (2022) 24:e28724. doi: 10.2196/28724, PMID: 35348462 PMC9006130

[19] Wicki S Clark IC Amann M Christ SM Schettle M Hertler C. Acceptance of digital health technologies in palliative care patients. Palliative Med Rep. (2024) 5:34–42. doi: 10.1089/pmr.2023.0062, PMID: 38249831 PMC10797306

[20] Schunn FA El Shafie RA Kronsteiner D Sauer LD Kudak A Bougatf N. Oncologic treatment support via a dedicated mobile app: a prospective feasibility evaluation (OPTIMISE-1). Strahlenther Onkol. (2024) 200:475–86. doi: 10.1007/s00066-023-02166-7, PMID: 37947806 PMC11111550

[21] Sprave T Pfaffenlehner M Stoian R Christofi E Rühle A Zöller D. App-controlled treatment monitoring and support for patients with head and neck cancer undergoing radiotherapy: results from a prospective randomized controlled trial. J Med Internet Res. (2023) 25:e46189. doi: 10.2196/46189, PMID: 37856185 PMC10623226

[22] Cho Y Zhang H Harris MR Gong Y Smith EL Jiang Y. Acceptance and use of home-based electronic symptom self-reporting systems in patients with cancer: systematic review. J Med Internet Res. (2021) 23:e24638. doi: 10.2196/24638, PMID: 33709929 PMC7998328

[23] Jagemann I Wensing O Stegemann M Hirschfeld G. Acceptance of medical artificial intelligence in skin cancer screening: choice-based conjoint survey. JMIR Form Res. (2024) 8:e46402. doi: 10.2196/46402, PMID: 38214959 PMC10818228

[24] Yang K Zeng Z Peng H Jiang Y. Attitudes of chinese cancer patients toward the clinical use of artificial intelligence. PPA Volume. (2019) 13:1867–75. doi: 10.2147/PPA.S225952, PMID: 31802856 PMC6830378

[25] Fransen SJ Kwee TC Rouw D Roest C van Lohuizen QY Simonis FFJ. Patient perspectives on the use of artificial intelligence in prostate cancer diagnosis on MRI. Eur Radiol. (2024) 35(2):769–75. doi: 10.1007/s00330-024-11012-y, PMID: 39143247 PMC11782406

[26] Jutzi TB Krieghoff-Henning EI Holland-Letz T Utikal JS Hauschild A Schadendorf D. Artificial intelligence in skin cancer diagnostics: the patients’ Perspective. Front Med. (2020) 7:233. doi: 10.3389/fmed.2020.00233, PMID: 32671078 PMC7326111

[27] Nelson CA Pérez-Chada LM Creadore A Li SJ Lo K Manjaly P. Patient perspectives on the use of artificial intelligence for skin cancer screening: A qualitative study. JAMA Dermatol. (2020) 156:501. doi: 10.1001/jamadermatol.2019.5014, PMID: 32159733 PMC7066525

[28] Mehnert A Müller D Lehmann C Koch U. Die deutsche Version des NCCN Distress-Thermometers. Z für Psychiatrie Psychol und Psychotherapie. (2006) 54:213–23. doi: 10.1024/1661-4747.54.3.213

[29] Kroenke K Spitzer RL Williams JBW Löwe B. An ultra-brief screening scale for anxiety and depression: the PHQ–4. Psychosomatics. (2009) 50:613–21. doi: 10.1016/S0033-3182(09)70864-3, PMID: 19996233

[30] Fydrich T Geyer M Hessel A Sommer G Brähler E. Fragebogen zur Sozialen Unterstützung (F-SozU): Normierung an einer repräsentativen Stichprobe. Diagnostica. (1999) 45:212–6. doi: 10.1026//0012-1924.45.4.212

[31] Soellner R Huber S Reder M. The concept of eHealth literacy and its measurement: German translation of the eHEALS. J Media Psychology: Theories Methods Appl. (2014) 26:29–38. doi: 10.1027/1864-1105/a000104

[32] R Core Team. R: A Language and Environment for Statistical Computing. R Foundation for Statistical Computing (2024).

[33] Kishan AU Ma TM Lamb JM Casado M Wilhalme H Low DA. Magnetic resonance imaging-guided vs computed tomography-guided stereotactic body radiotherapy for prostate cancer: the MIRAGE randomized clinical trial. JAMA Oncol. (2023) 9:365–73. doi: 10.1001/jamaoncol.2022.6558, PMID: 36633877 PMC9857817

[34] Huddart R Hafeez S Griffin C Choudhury A Foroudi F Syndikus I. Dose-escalated adaptive radiotherapy for bladder cancer: results of the phase 2 RAIDER randomised controlled trial. Eur Urol. (2025) 87(1):60–70. doi: 10.1016/j.eururo.2024.09.006, PMID: 39379236

[35] Bürkle SL Kuhn D Fechter T Radicioni G Hartong N Freitag MT. A student trained convolutional neural network competing with a commercial AI software and experts in organ at risk segmentation. Sci Rep. (2024) 14:25929. doi: 10.1038/s41598-024-76288-y, PMID: 39472608 PMC11522297

[36] Ancona C Caroppo E Lellis PD. Digital solutions supporting the quality of life of European cancer patients and their caregivers: a systematic literature review. Health Technol. (2024) 15(2):243–72. doi: 10.1101/2024.06.18.24309065

[37] Adriaans DJ Dierick-van Daele AT van Bakel MJHM Nieuwenhuijzen GA Teijink JA Heesakkers FF. Digital self-management support tools in the care plan of patients with cancer: review of randomized controlled trials. J Med Internet Res. (2021) 23:e20861. doi: 10.2196/20861, PMID: 34184997 PMC8278296

[38] Haemmerle R Paludo J Haddad TC Pritchett JC. The growing role of digital health tools in the care of patients with cancer: current use, future opportunities, and barriers to effective implementation. Curr Oncol Rep. (2024) 26:593–600. doi: 10.1007/s11912-024-01534-5, PMID: 38652424

[39] Haehl E Rühle A Spohn S Sprave T Gkika E Zamboglou C. Patterns-of-care analysis for radiotherapy of elderly head-and-neck cancer patients: A trinational survey in Germany, Austria and Switzerland. Front Oncol. (2021) 11:723716. doi: 10.3389/fonc.2021.723716, PMID: 35047384 PMC8761738

[40] Reis M Reis F Kunde W. Influence of believed AI involvement on the perception of digital medical advice. Nat Med. (2024) 30(11):3098–100. doi: 10.1038/s41591-024-03180-7, PMID: 39054373 PMC11564086

[41] Lythreatis S Singh SK El-Kassar A-N. The digital divide: A review and future research agenda. Technol Forecasting Soc Change. (2022) 175:121359. doi: 10.1016/j.techfore.2021.121359

[42] Goerling U Ernst J Esser P Haering C Hermann M Hornemann B. Estimating the prevalence of mental disorders in patients with newly diagnosed cancer in relation to socioeconomic status: a multicenter prospective observational study. ESMO Open. (2024) 9:103655. doi: 10.1016/j.esmoop.2024.103655, PMID: 39088984 PMC11345380

[43] Chang T-G Cao Y Sfreddo HJ Dhruba SR Lee S-H Valero C. LORIS robustly predicts patient outcomes with immune checkpoint blockade therapy using common clinical, pathologic and genomic features. Nat Cancer. (2024) 5:1158–75. doi: 10.1038/s43018-024-00772-7, PMID: 38831056 PMC11962634

[44] Dima A Nabergoj‐Makovec U van Boven JM. Digital tools and medication adherence. In: Elseviers M Wettermark B Benkó R, editors. Drug Utilization Research, 2nd ed. Wiley (2024). p. 419–27.

[45] Ronckers C Spix C Trübenbach C Katalinic A Christ M Cicero A. Krebs in Deutschland für 2019/2020. (2023). doi: 10.25646/11357

