# Supplementary material for: Digital support and artificial intelligence in cancer patients undergoing radiation therapy: patient utilization, acceptance and attitudes
Source: Front Oncol. 2025 Sep 26;15:1546221. doi: 10.3389/fonc.2025.1546221 (PMC12510812; doi:10.3389/fonc.2025.1546221)
Supplement: Supplementary file 1 [file Table1.docx]

**SUPPLEMENTARY MATERIAL**

| **Table S1.** Items on Utilization, Acceptance and Attitudes toward Artificial Intelligence and Digital Support Tools (**German** original version in bold and *English* translation in cursive) | | | | | | | | | | | | | | | | | | | | | |
| --- | --- | --- | --- | --- | --- | --- | --- | --- | --- | --- | --- | --- | --- | --- | --- | --- | --- | --- | --- | --- | --- |
| **1.** | | | **Haben Sie sich schon einmal für die Nutzung einer telemedizinischen Versorgung (Versorgung per Telefon oder Internet) entschieden?** *Have you ever used telehealth services (care by telephone or internet)?* | | | | | | | | | | | | | | | | | | |
|  | | | **🔿_0_** | | **Nein**  *No* | | | **🔿_1_** | | **Ja** *Yes* | | | | | | | | | | | |
| 2. | | **Wie oft haben Sie in den letzten 12 Monaten das Internet genutzt, um Informationen oder Empfehlungen über Ihre Gesundheit zu suchen?** *How often have you used the internet in the last 12 months to search for information or advice about your health?* | | | | | | | | | | | | | | | | | | | |
|  | | **🔿_1_** | | | **Weniger als einmal pro Monat** *Less than once a month* | | | | | | | | | | **🔿_3_** | | **Mehrmals wöchentlich** *Several times a week* | | | | |
|  | | **🔿_2_** | | | **Mehrmals im Monat** *Several times a month* | | | | | | | | | | **🔿_4_** | | **Täglich** *Daily* | | | | |
| **3.** | | **Ich bin neuen Technologien (Telemedizin, Künstlicher Intelligenz, etc.) bei meiner medizinischen Versorgung gegenüber grundsätzlich …** *To new technologies (telemedicine, artificial intelligence, etc.) in my healthcare I am generally…* | | | | | | | | | | | | | | | | | | | |
|  | | **🔿_0_** | | | | | **🔿_1_** | | | **🔿_2_** | | **🔿_3_** | **🔿_4_** | **🔿_5_** | | **🔿_6_** | | **🔿_7_** | **🔿_8_** | **🔿_9_** | **🔿_10_** |
|  |  | **Gar nicht aufgeschlossen** *Not open at all* | | | | | | | |  | |  |  |  | |  | |  | **Sehr  aufgeschlossen** *Very open* | | |
| **4.** | **Ich habe Vertrauen in neue Technologien (Telemedizin, Künstliche Intelligenz, etc.) für meine gesundheitliche Versorgung.** *I trust in new technologies (telemedicine, artificial intelligence, etc.) for my healthcare.* | | | | | | | | | | | | | | | | | | | | |
|  | **🔿_0_** | | | | | **🔿_1_** | | | | **🔿_2_** | | **🔿_3_** | **🔿_4_** | **🔿_5_** | | **🔿_6_** | | **🔿_7_** | **🔿_8_** | **🔿_9_** | **🔿_10_** |
|  | **Gar nicht** *Not at all* | | | | | | | | |  | |  |  |  | |  | |  |  | **Sehr** *Very much* | |
| **5.** | **Wären Sie bereit Gesundheitsinformationen mit Ihrem medizinischen Behandlungsteam digital zu teilen und auszutauschen?** *Would you be willing to digitally share health information with your healthcare professionals?* | | | | | | | | | | | | | | | | | | | | |
|  | **🔿_0_** | | | **Nein** *No* | | | | | **🔿_1_** | | **Ja** *Yes* | | | | | | | | | | |

| **Künstliche Intelligenz (KI) wird in der medizinischen Behandlung von Krebs bereits vielfältig eingesetzt. Durch den Einsatz von KI können Ärzte genaue Diagnosen stellen und auf den Patienten abgestimmte Therapien entwickeln, um die Behandlung zu verbessern. KI-Systeme können zum Beispiel große Mengen an medizinischen Bildern und Patientendaten analysieren, um in der Strahlentherapie Tumore frühzeitig zu erkennen oder individuelle Strahlenpläne zu erstellen. Darüber hinaus helfen KI-Systeme Ärzten auch bei der Interpretation von Bildern wie MRT- oder CT-Scans, indem sie Auffälligkeiten hervorheben oder Empfehlungen für weitere Untersuchungen geben.** *Artificial intelligence (AI) is already being used in a variety of ways in the medical treatment of cancer. By using AI, doctors can make accurate diagnoses and develop customized therapies to improve treatment. For example, AI systems can analyze large data of medical images and patient data in order to detect tumors early in radiotherapy or to create individual radiotherapy plans. In addition, AI systems also help doctors to interpret images such as MRI or CT scans by highlighting abnormalities or making recommendations for further examinations.* | | | | | | | | | | | | |
| --- | --- | --- | --- | --- | --- | --- | --- | --- | --- | --- | --- | --- |
| **6.** | | | **Fühlen Sie sich über die Möglichkeiten des Einsatzes von KI in Ihrer Strahlenbehandlung ausreichend informiert?** *Do you feel sufficiently informed about the possibilities of using AI in your radiotherapy treatment?* | | | | | | | | | |
|  | | | **🔿_0_** | | **Nein**  *No* | **🔿_1_** | **Ja** *Yes* | | | | | |
| **7.** | | **Würden Sie dem Einsatz von KI in Ihrer Strahlenbehandlung während der Diagnostik und Therapieplanung zustimmen?** Would you agree to the use of AI in your radiotherapy treatment during diagnostics and treatment planning? | | | | | | | | | | |
|  | | **🔿_0_** | | **Nein**  *No* | | **🔿_1_** | **Ja** *Yes* | | | | | |
| **8.** | | **Wem würden Sie mehr vertrauen: einer Behandlung und Auswertung von Laborergebnissen  durch einen Arzt oder durch ein gut etabliertes KI-System?** *Who would you trust more: treatment and analysis of laboratory results by a doctor or by a well-established AI system?* | | | | | | | | | | |
|  | | **🔿_1_** | | **Behandlung durch den Arzt** *Treatment by the doctor* | | | | | | | | |
|  | | **🔿_2_** | | **Behandlung durch ein gut etabliertes KI-System** *Treatment by a well-established AI system* | | | | | | | | |
|  | | **🔿_3_** | | **Behandlung durch den Arzt in Kombination mit einem gut etablierten KI-System** *Treatment by the doctor in combination with a well-established AI system* | | | | | | | | |
| **9.** | **Wir sind an Ihrer Meinung zu KI in der Strahlenbehandlung und der allgemeinen medizinischen Behandlung interessiert. Bitte geben Sie an, inwiefern Sie den folgenden Aussagen zustimmen.** *We are interested in your opinion on AI in radiotherapy and general medical treatment. Please indicate to what extent you agree with the following statements.* | | | | | | | | | | | |
|  | | | | | | | | **stimme überhaupt nicht zu** *strongly disagree* | **stimme nicht zu** *disagree* | **neutral** *neutral* | **stimme  zu** *agree* | **stimme stark zu** *strongly agree* |
| **Ich weiß nicht, wie KI in der Strahlenbehandlung funktioniert.** *I don't know how AI works in radiotherapy.* | | | | | | | | **🔿_1_** | **🔿_2_** | **🔿_3_** | **🔿_4_** | **🔿_5_** |
| **Ich denke, KI trifft schlechtere Entscheidungen als mein Arzt, da dieser besser weiß was gut für mich ist.** *I think AI makes worse decisions than my doctor because they know better what is good for me.* | | | | | | | | **🔿_1_** | **🔿_2_** | **🔿_3_** | **🔿_4_** | **🔿_5_** |
| **Ich lehne technologische Lösungen und KI generell ab.** *I generally reject technological solutions and AI.* | | | | | | | | **🔿_1_** | **🔿_2_** | **🔿_3_** | **🔿_4_** | **🔿_5_** |
| **Ich habe Angst vor möglichen Fehlern der KI.** *I am afraid of possible mistakes by the AI.* | | | | | | | | **🔿_1_** | **🔿_2_** | **🔿_3_** | **🔿_4_** | **🔿_5_** |
| **Der Einsatz von KI ist mir unheimlich.** *The use of AI scares me.* | | | | | | | | **🔿_1_** | **🔿_2_** | **🔿_3_** | **🔿_4_** | **🔿_5_** |

| **Digitale Unterstützungsangebote während einer Krebserkrankung sollen Patienten helfen, besser mit ihrer Krankheit und den möglichen Folgen umzugehen. Dazu gehören zum Beispiel die Möglichkeit selbst Symptome der Nebenwirkungen digital zu erfassen, Informationsmaterial über Krebs auf Websites oder in Apps, Online-Foren mit anderen Patienten, das Überwachen von Körperfunktionen wie Puls und Blutdruck mithilfe von Smartwatches und vieles mehr.** *Digital support tools during a cancer disease should help patients to cope better with their illness and the potential side effects. These include, for example, the ability to digitally assess symptoms of side effects, information material about cancer on websites or apps, online forums with other patients, monitoring bodily functions such as pulse and blood pressure using smartwatches and much more.* | | | | | | | | | | | | | | | | | | |
| --- | --- | --- | --- | --- | --- | --- | --- | --- | --- | --- | --- | --- | --- | --- | --- | --- | --- | --- |
| 10. | | | | | **Welche digitalen Unterstützungsangebote nutzen Sie aktuell bzw. haben Sie in der Vergangenheit genutzt?  Bitte kreuzen Sie alle zutreffenden Antworten an und geben Sie, falls möglich, die genaue Bezeichnung der Angebote an.** *Which digital support services do you currently use or have you used in the past?  Please select all applicable answers and, if possible, give the exact name of the services.* | | | | | | | | | | | | | |
|  | | | | | **🔿_1_** | | | | | **Webseiten:** *Websites: ____________________________________________________________________________* | | | | | | | | |
|  | | | | | **🔿_2_** | | | | | **Apps:** *Apps: ________________________________________________________________________________* | | | | | | | | |
|  | | | | | **🔿_3_** | | | | | **Telemedizin (z.B. online Arztbesuche, Terminvereinbarung):** *Telehealth (e.g. online doctors' consultations, making appointments: _____________________________* | | | | | | | | |
|  | | | | | **🔿_4_** | | | | | **Soziale Medien, Online-Selbsthilfegruppen:** *Social Media, Online support groups: ______________________________________________________* | | | | | | | | |
|  | | | | | **🔿_5_** | | | | | **Gesundheitsapps für Smartwatches (z.B. Fitness-Tracker):** *Healthapps für wearables (e.g. fitness tracker): ______________________________________________* | | | | | | | | |
|  | | | | | **🔿_6_** | | | | | **Sonstiges:** *Other: _______________________________________________________________________________* | | | | | | | | |
|  | | | | | | **Wenn Sie digitale Unterstützungsangebote nutzen, aus welchen Gründen? Bitte kreuzen Sie alle zutreffenden Antworten an.** *If you use digital support tools, for what reasons? Please select all that apply.* | | | | | | | | | | | | |
|  | | | | | | **🔿_1_** | | | | | **Ich erhalte Informationen über meine Krankheit und Behandlung.** *I receive information about my illness and treatment.* | | | | | | | |
|  | | | | | | **🔿_2_** | | | | | **Ich kann Nebenwirkungen, Symptome und Körperfunktionen eingeben und kontrollieren.** *I can assess and monitor side effects, symptoms and bodily functions.* | | | | | | | |
|  | | | | | | **🔿_3_** | | | | | **Ich kann mich mit anderen Patienten austauschen.** *I can talk to other patients.* | | | | | | | |
|  | | | | | | **🔿_4_** | | | | | **Ich bekomme Tipps zur Bewältigung körperlicher Begleiterscheinungen.** *I receive information on how to cope with physical side effects.* | | | | | | | |
|  | | | | | | **🔿_5_** | | | | | **Ich bekomme Tipps zur Bewältigung psychischer Begleiterscheinungen.** *I receive information on how to cope with psychological side effects.* | | | | | | | |
|  | | | | | | **🔿_6_** | | | | | **Ich erhalte Empfehlungen für einen gesunden Lebensstil.** *I receive recommendations for a healthy lifestyle.* | | | | | | | |
|  | | | | | | **🔿_7_** | | | | | **Ich möchte medizinische Informationen online mit meinem Behandlungsteam teilen (z.B. Symptome, Körperfunktionen).** *I would like to share medical information online with my treatment team (e.g. symptoms, bodily functions).* | | | | | | | |
|  | | | | | | **🔿_8_** | | | | | **Sonstiges:** *Other:* | | | | | | | |
|  | | | | | | **Wenn Sie keine digitale Unterstützungsangebote nutzen, aus welchen Gründen? Bitte kreuzen Sie alle zutreffenden Antworten an.** *If you don’t use digital support tools, for what reasons? Please select all that apply.* | | | | | | | | | | | | |
|  | | | | | | **🔿_1_** | | | | | **Ich möchte lieber persönlichen Kontakt mit meinem Arzt.** *I prefer personal contact with my doctor.* | | | | | | | |
|  | | | | | | **🔿_2_** | | | | | **Ich habe Datenschutz- und Sicherheitsbedenken.** *I have concerns about data protection and security.* | | | | | | | |
|  | | | | | | **🔿_3_** | | | | | **Ich habe Schwierigkeiten, mit der Technik umzugehen.** *I have difficulties handling the technology.* | | | | | | | |
|  | | | | | | **🔿_4_** | | | | | **Es sind keine guten Angebote vorhanden.** *There are no good services available.* | | | | | | | |
|  | | | | | | **🔿_5_** | | | | | **Sonstiges:** *Other:* | | | | | | | |
| 11. | | | | **Wie oft nutzen Sie digitale Unterstützungsangebote?** *How often do you use digital support tools?* | | | | | | | | | | | | | | |
|  | | | | **🔿_1_** | | | | | **Weniger als einmal pro Monat** *Less than once a month* | | | **🔿_3_** | **Mehrmals wöchentlich** *Several times a week* | | | | | |
|  | | | | **🔿_2_** | | | | | **Mehrmals im Monat** *Several times a month* | | | **🔿_4_** | **Täglich** *Daily* | | | | | |
| 12. | | | **Wo suchen Sie am liebsten Informationen und Unterstützung bei körperlichen Begleit­sympto­men?** *Where do you prefer to look for information and support for physical side effects?* | | | | | | | | | | | | | | | |
|  | | | **🔿_1_** | | | | | **Persönlich bei meinem Arzt oder medizinischen Behandlungsteam** *In person with my doctor or health care professionals* | | | | | | | | | | |
|  | | | **🔿_2_** | | | | | **Im Internet (Webseiten, Foren, Apps)** *Online (websites, forums, apps)* | | | | | | | | | | |
|  | | | **🔿_3_** | | | | | **Sonstiges:** *Other: _______________________________________________________________________________* | | | | | | | | | | |
| 13. | | **Wo suchen Sie am liebsten Informationen und Unterstützung bei psychischen Begleitsymptomen?** *Where do you prefer to look for information and support for psychological side effects?* | | | | | | | | | | | | | | | | |
|  | | **🔿_1_** | | | | | **Persönlich bei meinem Arzt oder medizinischen Behandlungsteam** *In person with my doctor or health care professionals* | | | | | | | | | | | |
|  | | **🔿_2_** | | | | | **Im Internet (Webseiten, Foren, Apps)** *Online (websites, forums, apps)* | | | | | | | | | | | |
|  | | **🔿_3_** | | | | | **Sonstiges:** *Other: _______________________________________________________________________________* | | | | | | | | | | | |
| **14.** | **Wir sind an Ihrer Meinung zu digitalen Unterstützungsangeboten interessiert. Bitte geben Sie an, inwiefern Sie den folgenden Aussagen zustimmen.** *We are interested in your opinion on digital support tools. Please indicate the extent to which you agree with the following statements.* | | | | | | | | | | | | | | | | | |
|  | | | | | | | | | | | | | | **stimme überhaupt nicht zu** *strongly disagree* | **stimme nicht zu** *disagree* | **neutral** *neutrak* | **stimme  zu** *agree* | **stimme stark zu** *strongly agree* |
| **Digitale Unterstützungsangebote haben mir geholfen, Entscheidungen über meine Krebserkrankung zu treffen, zum Beispiel in Bezug auf Behandlungen oder den Umgang mit Nebenwirkungen.** *Digital support tools have helped me to make decisions about my cancer disease, for example in relation to treatments or dealing with side effects.* | | | | | | | | | | | | | | **🔿_1_** | **🔿_2_** | **🔿_3_** | **🔿_4_** | **🔿_5_** |
| **Digitale Unterstützungsangebote sind eine sinnvolle Ergänzung zu persönlichen Unterstützungs­angeboten.** *Digital support tools are a valuable addition to in-person support services.* | | | | | | | | | | | | | | **🔿_1_** | **🔿_2_** | **🔿_3_** | **🔿_4_** | **🔿_5_** |
| **Digitale Unterstützungsangebote sind nur für junge Patienten geeignet.** *Digital support services are only suitable for young patients.* | | | | | | | | | | | | | | **🔿_1_** | **🔿_2_** | **🔿_3_** | **🔿_4_** | **🔿_5_** |
| **Körperliche und psychische Begleitsymptome können nur persönlich durch das medizinische Behandlungs­team verbessert werden (z.B. Medikamente, Arzt­gespräche, Psychotherapie).** *Physical and psychological side effects can only be treated in-person by healthcare professionals (e.g. medication, medical consultations, psychotherapy).* | | | | | | | | | | | | | | **🔿_1_** | **🔿_2_** | **🔿_3_** | **🔿_4_** | **🔿_5_** |
| **Ärzte sollten digitale Unterstützungsangebote standardmäßig empfehlen.** *Doctors should routinely recommend digital support tools.* | | | | | | | | | | | | | | **🔿_1_** | **🔿_2_** | **🔿_3_** | **🔿_4_** | **🔿_5_** |
| **Ich kenne die digitalen Unterstützungsangebote, die  mir im Rahmen meiner Krebserkrankung zur Verfügung stehen, und fühle mich ausreichend informiert.** *I know the digital support services that are available to me in the context of my cancer and feel sufficiently informed.* | | | | | | | | | | | | | | **🔿_1_** | **🔿_2_** | **🔿_3_** | **🔿_4_** | **🔿_5_** |
